# Assessment of *in vivo* epithelial disruption to model anorectal *Chlamydia* infection in mice

**DOI:** 10.64898/2026.10.24.761026

**Published:** 2026-10-27

**Authors:** Zoe E.R. Wilton, Susan B. Core, Leslie M. Huggins, Andzoa N Jamus, Isabella G. Romano, James P. Davis, Anthony D. Cretara, Kathryn M. Frietze

## Abstract

*Chlamydia trachomatis* (Ct) causes the most commonly diagnosed bacterial sexually transmitted infection. Ct can infect mucosal epithelial cells throughout the human body and is frequently found at the anorectal tract. However, there are currently few models of anorectal *Chlamydia* infection, limiting our ability to investigate vaccines, identify therapeutics, and understand infection dynamics. Since epithelial disruption prior to infection has been used in other STI animal models to increase permissibility and improve reproducibility of infection, we evaluated the impact of different methods of epithelial disruption prior to inoculation on the bacterial burden of anorectal *Chlamydia muridarum* (Cm). Mice were treated anorectally with physical (calcium alginate swab) and/or chemical (nonoxynal-9) disruption 24 hours prior to anorectal inoculation with 1 x 10 ^4^ IFUs of a luciferase-expressing Cm (Luc-Cm). Infection progression was monitored in real time using an *in vivo* imaging system (IVIS) and the shedding of Luc-Cm DNA in the stool was quantified by qPCR. Cm was detected in all infected treatment groups by both IVIS and qPCR, irrespective of treatment. However, N-9 treatment prior to inoculation with Luc-Cm increased infection reproducibility and persistence when monitored using IVIS imaging, while having minimal adverse reactions. Therefore, chemical disruption prior to Luc-Cm infection was utilized to examine the efficacy of a bacteriophage virus-like particle (VLP) vaccine against Luc-Cm (Qβ-VD4-Cm) in the anorectal tract. We found that immunization with Qβ-VD4-Cm did not reduce Luc-Cm infection in the anorectal tract, despite this vaccine previously being shown to reduce Luc-Cm infection in the female reproductive tract. This highlights the unique nature of vaccine-mediated protection that can occur at distinct anatomical sites of infection with the same pathogen.

## Full Text Availability

The license terms selected by the author(s) for this preprint version do not permit archiving in PMC. The full text is available from the preprint server.

